# *PLAC1* Expression Decreases in Chorionic Villi in Response to Labor

**DOI:** 10.1155/2013/704252

**Published:** 2013-06-11

**Authors:** Yahdira M. Rodriguez-Prado, Xiaoyuan Kong, Michael E. Fant

**Affiliations:** ^1^Department of Pediatrics, University of South Florida Morsani College of Medicine, Tampa, FL 33606, USA; ^2^Department of Obstetrics and Gynecology, University of South Florida Morsani College of Medicine, Tampa, FL 33606, USA; ^3^Department of Pathology and Cell Biology, University of South Florida Morsani College of Medicine, Tampa, FL 33606, USA

## Abstract

*PLAC1* (Placenta-Specific 1) is a recently described, trophoblast-expressed gene essential for normal placental development. The protein localizes to the microvillus membrane surface of the syncytiotrophoblast in direct proximity to the maternal compartment. Although its role has not been defined, increased circulating levels of human *PLAC1* mRNA in maternal blood are associated with preeclampsia. Furthermore, *PLAC1*-null mice exhibit decreased viability in the peripartum period suggesting a role in pregnancy maintenance late in gestation. We examined *PLAC1* gene expression in the human placenta during normal pregnancy and pregnancies associated with maternal diabetes and preeclampsia using quantitative, real time PCR (q-RT-PCR). Although there was no apparent difference in *PLAC1* gene expression among human pregnancies complicated by diabetes or preeclampsia, an unexpected effect of labor was noted at term. *PLAC1* expression in placentae delivered vaginally following induced or spontaneous labor was significantly reduced compared to placentae not exposed to labor making it one of only a few placental genes influenced by labor. The significance of this finding is unknown. Viewed in the context of its importance in placental development, however, these findings are consistent with a role for *PLAC1* in the maintenance of the maternal-fetal interface.

## 1. Introduction

*PLAC1* is a recently identified X-linked gene [1]. Compared to normal adult tissues, its expression is restricted primarily to cells of trophoblast lineage. The putative PLAC1 protein contains a signal peptide and a ZP3 motif (zona pellucida 3) suggesting it targets the secretory pathway and likely interacts with other membrane-associated proteins [2, 3]. Subsequent studies have confirmed its localization to the maternal surface of the syncytiotrophoblast, placing it in direct contact with the maternal compartment, suggesting it may be involved in protein interactions at the maternal-fetal interface [4]. 

 Several studies have identified *PLAC1* as a potential biomarker for gestational pathologies relevant to human health. Farina et al. (2005) first demonstrated that circulating *PLAC1* mRNA in maternal blood was diminished in pregnancies associated with threatened abortion prior to 20 weeks gestation [5]. Subsequently, elevated levels of circulating *PLAC1* mRNA were observed in pregnancies complicated by preeclampsia and were directly related to disease severity [6, 7]. Recently, we reported that women can become sensitized to the PLAC1 antigen during pregnancy and the presence of anti-PLAC1 antibodies may be associated with infertility and/or recurrent pregnancy loss [8]. This observation was later supported by Matteo et al. who demonstrated increased titers of anti-PLAC1 antibodies in women with a history of infertility [9]. 

 Using a mutant mouse model we have recently confirmed that *PLAC1* is essential for normal placental development. The absence of *PLAC1* results in marked placentomegaly and mild intrauterine growth retardation indicating some degree of placental insufficiency [10]. While the *PLAC1* knockout (KO) is not lethal, it is associated with decreased viability. The distribution of genotypes among viable prenatal and postnatal progeny indicates that something occurs late in gestation to increase the probabilistic risk of perinatal death. Collectively, these observations suggest that *PLAC1* contributes, in part, to regulatory processes at the maternal-fetal interface and may be particularly important for fetal survival late in gestation and/or during parturition. We therefore sought to examine *PLAC1* expression during human gestation in the presence or absence of common gestational disorders associated with adverse fetal outcomes. 

## 2. Materials and Methods

### 2.1. Study Subjects

 Placental tissue was collected from women delivered at Memorial Hermann Hospital (Houston, Texas) and Tampa General Hospital (Tampa, FL) under protocols approved by the Institutional Review Boards of the University of South Florida and the University of Texas-Houston Medical School in accordance with the Code of Ethics of the World Medical Association (Declaration of Helsinki) for experiments involving humans. Initially, placental samples were obtained at various gestational ages from normal pregnancies or those complicated by the clinical diagnoses of diabetes and/or preeclampsia. Although preterm pregnancies are inherently not normal, “control” pregnancies throughout gestation were defined as the absence of known maternal disease, intrauterine growth restriction (IUGR), congenital anomalies, multiple gestations, chromosomal defects, and clinical chorioamnionitis.

 Subsequently, placental samples were obtained to specifically examine the effects of labor on *PLAC1* expression. Samples were obtained from normal, term human placentae (38–41 weeks gestation) immediately after delivery by elective C/S (in the absence of labor) or vaginally (in the presence of labor). Labor was further delineated into groups associated with the spontaneous onset of labor (SVD) and induced labor (IVD). Exclusion criteria for these patients included multiple births, diabetes, intrauterine growth retardation, preeclampsia, hypertension, diabetes, autoimmune disease, placental insufficiency, and infection.

### 2.2. Tissue Collection

 A total of 3 × 0.5 cm. samples were obtained from different sites in each placenta (near the umbilical cord, at the periphery and midway between these two points) and pooled. Samples were rinsed in PBS to remove maternal blood, placed in RNAlater (Ambion/Life Technologies, Carlsbad, CA), and stored at −20°C until RNA isolation. 

### 2.3. Quantitative, Real-Time PCR

 After disruption and homogenization of tissue specimens, total RNA was extracted using the AllPrep DNA/RNA Mini Kit (Qiagen, Valencia, CA). 2 *μ*g of total RNA was used to synthesize the complementary cDNA with primer oligo dT and the SuperScript III First-Strand Synthesis SuperMix (Invitrogen/Life Technologies, Carlsbad, CA) according to the manufacturer's protocol. The RT-PCR reaction was performed using 2 *μ*L cDNA with 1 *μ*L 20X Taqman Human *PLAC1* probe (Assay ID number Hs00222307_m1) (Life Technologies, Carlsbad, CA), 10 *μ*L TaqMan Universal PCR Master Mix (Life Technologies, Carlsbad, CA) and 7 *μ*L autoclaved RNAse free water for a total volume of 20 *μ*L. As an internal control, a second RT-PCR reaction was performed using 2 *μ*L of a 1 : 10 diluted cDNA with 1 *μ*L 20X Taqman 18S probe (Assay ID number Hs99999901_s1) (Life Technologies, Carlsbad, CA). 10 *μ*L 2X Master Mix and 7 *μ*L autoclaved RNAse-free water for a total volume of 20 *μ*L. Thermocycling conditions were as follows: 50°C, 2 min; 95°C, 10 min; and 40 cycles of 95°C for 15 s and 60°C for 1 min. Human *PLAC1* mRNA expression relative to 18S ribosomal RNA was calculated. Each sample was run in triplicate.

### 2.4. Statistical Analyses

 Statistical analyses were performed using analysis of variance (ANOVA) and the independent *t*-test.

## 3. Results

**Figure 1:**
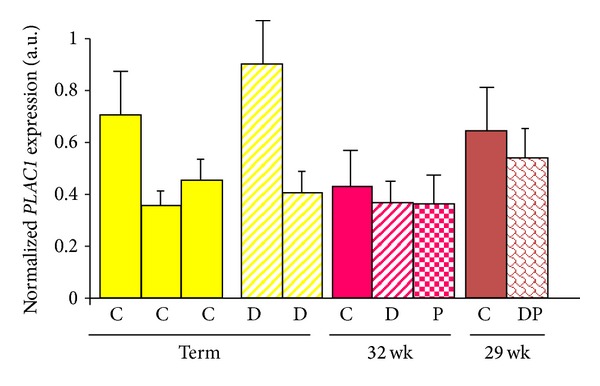
*PLAC1* mRNA expression in placentae associated with normal and abnormal pregnancies. PLAC1 mRNA expression was measured by quantitative, real-time PCR and normalized to 18S ribosomal RNA. Each bar represents the mean + SD (standard deviation) of triplicate samples obtained from a single placenta. Each sample was run in triplicate. C = normal pregnancy; D = maternal diabetes; P = preeclampsia; DP = presence of both diabetes and preeclampsia.

 Preliminary examination of placentae obtained at various gestational ages revealed no discernable effect of diabetes or preeclampsia on *PLAC1* expression (Figure 1). However, higher *PLAC1* expression was noted in one control and one diabetic placenta at term compared to the other age-matched control and diabetic placentae. Review of the clinical history associated with each placenta revealed that the control and diabetic placentae expressing higher levels of *PLAC1 *were delivered via scheduled C-section (in the absence of labor) suggesting that *PLAC1* expression may be influenced by labor. 

**Figure 2:**
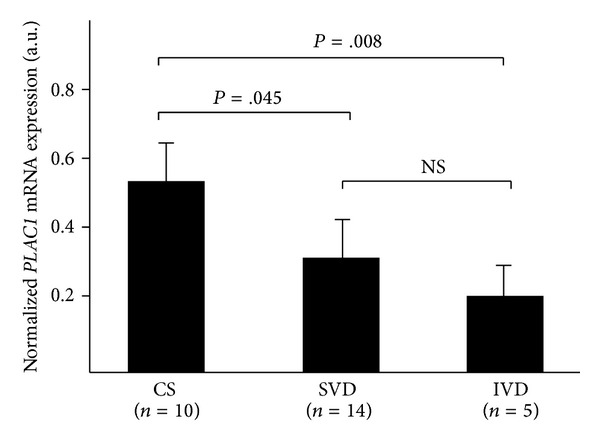
*PLAC1* mRNA expression in chorionic villi is influenced by the presence of labor. *PLAC1* mRNA expression was measured by quantitative, real-time PCR and expressed as a function of 18S ribosomal RNA. *PLAC1* expression is significantly lower in placentae delivered vaginally after the spontaneous onset of labor (SVD) or induced labor (IVD) compared to placentae delivered via elective C/S in the absence of labor. Error bars = standard deviation; NS = not significant.

 In order to examine the possibility that *PLAC1* expression was influenced by labor we obtained placental tissue from uncomplicated term pregnancies that delivered in the presence or absence of labor. Placentae assigned to each group (C/S, SVD, IVD) were associated with pregnancies that did not differ significantly in gestational age, birth weight, or Apgar scores (Table 1). As shown in Figure 2, *PLAC1 *mRNA expression was significantly lower in placentae delivered vaginally after the spontaneous (SVD) or induced (IVD) onset of labor compared to placentae delivered via elective C/S in the absence of labor. Statistical analyses indicated a highly significant difference between C/S deliveries and either SVD or IVD, but no difference in expression between the SVD and IVD groups. 

## 4. Discussion

 We have examined the expression of *PLAC1* gene expression during normal human pregnancies and pregnancies associated with maternal diabetes and preeclampsia. Although no significant associations with maternal diabetes or preeclampsia were observed, we were able to demonstrate that its expression is attenuated in response to labor. Previous studies have attempted to identify changes in placental gene expression during labor. A gene expression profile representing 24,650 human genes and 37,123 gene transcripts found 92 placental genes to be downregulated and 94 genes to be upregulated by labor. However, none of these genes was differentially expressed to a significant degree, that is, greater than 2-fold [11]. More recently, Lee et al. [12] found 351 transcripts to be differentially expressed in the vaginal delivery group compared to nonlabored, C/S group. Of these genes, 344 genes were upregulated and only 7 were downregulated. The affected genes tended to represent functions related to oxidative stress, angiogenesis, and cell death. As indicated by the above studies, the total number of placental genes influenced by labor comprises an extremely small percentage of the total number of expressed genes supporting the notion that the reduced *PLAC1* expression reflects its important role in pregnancy maintenance and/or parturition. Alternatively, it may simply reflect a nonspecific response to the inflammation, oxidative stress, or hypoperfusion associated with labor. These possibilities seem less likely due to the lack of evidence pointing to a more global disruption of gene expression.

 Insights into potential transcriptional mechanisms involved in reduced *PLAC1* expression are offered by studies demonstrating that PPAR-*α*, *δ*, *γ* and RXR-*α* expression by placenta and choriodecidua are influenced by labor [13, 14]. The implications of these findings are compelling given the importance of PPARs and RXR-*α* to placental development and uterine quiescence [15, 16]. The relevance of labor-associated changes in PPAR and RXR-*α* expression to *PLAC1* was provided by Chen et al. [17]. Two *PLAC1* promoters were identified that utilize RXR/LXR to drive *PLAC1* expression. PPARs form heterodimers with RXR-*α*. It is possible that altered RXR/PPAR expression occurring in response to labor influences *PLAC1* expression at the transcriptional level. However, this remains to be validated experimentally.

 In summary we have shown that *PLAC1, *a gene essential for normal placental development, is one of only a few placental genes exhibiting reduced expression during labor. These studies do not necessarily implicate *PLAC1* in the initiation of labor or parturition and a specific link should be viewed cautiously. However, they are consistent with a role for PLAC1 in regulating syncytiotrophoblast function at its apical, maternal-facing membrane surface. The physiological significance of this observation will be clarified as the protein interactions that underlie the mechanism of PLAC1 action are delineated in future studies.

## 5. Conclusions

*PLAC1* gene expression is significantly decreased after the onset of labor in the human placenta. The significance of this observation is not yet known. However, its robust expression by cells of trophoblast lineage, its requirement for normal placental development, and the paucity of other placental genes affected by labor are consistent with the hypothesis that PLAC1 acts at the apical surface of the syncytiotrophoblast to support regulatory processes relevant to the maintenance of the maternal-fetal interface. The basis for the interaction between labor and *PLAC1* expression awaits further studies. 

## Figures and Tables

**Table 1:** Characteristics of study groups.

	SVD	IVD	C/S
Number	14	5	10
Gestational age (weeks)	38.8	40	39.1
Birth weight (gm) ± SD	3256 ± 363	3416 ± 740	3338 ± 489
Apgar-1 min	9	8.2	8.9
Apgar-5 min	9	9	9
Duration of rupture (min) ± SD	386 ± 350	701 ± 1293	1

SD: standard deviation.
